# A Parametric Study of Droplet Directional Detachment on Inverted Wedge Patterns with Wettability Contrast

**DOI:** 10.3390/mi17050575

**Published:** 2026-05-07

**Authors:** Dalong Liang, Wenbin Cui

**Affiliations:** Marine Engineering College, Dalian Maritime University, Dalian 116026, China; cuiwenbin@dlmu.edu.cn

**Keywords:** droplet detachment, wedge pattern, wettability, liquid dynamic behavior

## Abstract

Constructing inverted wedge-shaped hydrophilic channels with a small apex angle on surfaces with wettability patterns is an effective strategy to promote efficient and complete droplet detachment, which is crucial for applications such as condensation heat transfer and self-cleaning. However, a comprehensive understanding of how wedge geometry parameters affect droplet dynamics has not been established. In this study, we systematically investigate the dynamics of droplet formation and detachment within inverted wedge-shaped superhydrophilic channels fabricated by laser etching on hydrophobic or superhydrophobic substrates. Four distinct droplet detachment mechanisms are revealed. Our results indicate that, within the experimental parameters tested, a slender channel geometry—featuring a narrow upper base, a minimized lower base, and sufficient height—combined with a superhydrophobic substrate, promotes high-position droplet formation, extends the droplet sliding distance, and significantly reduces resistance. This synergy leads to the most efficient detachment mechanism: inertia-driven direct shedding. For the tested configurations, the C1.2/0/40 channel achieved the highest recorded detachment frequency of 318 min^−1^ at a flow rate of 0.5 mL/min. Furthermore, droplet rebound at the channel tip is observed in some configurations, where two to three droplets must form sequentially and coalesce to trigger a single detachment event. This work provides actionable geometric design strategies for engineering surfaces capable of directional and highly efficient droplet detachment.

## 1. Introduction

The precise manipulation of droplet motion is crucial for diverse applications, including microfluidics [1], water harvesting [2], and enhanced condensation heat transfer [3]. While active driving methods (e.g., magnetic [4], electrical [5,6], thermal [7] or vibrational [8] actuation) provide precise control, passive strategies that exploit surface energy or structural gradients are frequently favored due to their inherent advantages in sustainability, potential for miniaturization, and operational simplicity [9]. Among these passive approaches, patterned surfaces with wettability gradients, such as Janus surfaces [10,11] or stripe patterns [12,13,14], have been widely investigated. Notably, “wedge-shaped” hydrophilic channels on hydrophobic or superhydrophobic substrates [15,16,17] have shown exceptional ability for spontaneous and directional liquid transport, often drawing inspiration from natural systems like cactus spines [18]. The mechanism has been well understood: a significant free energy gradient, resulting from a sharp wettability contrast and the wedge apex angle, creates a Laplace pressure difference that drives droplets from the narrow apex toward the wider opening of the channel [19].

This “pumping” function for liquid collection has been extensively studied, with research focusing on optimizing geometric parameters like apex angle and track length to maximize transport speed and distance [20,21,22,23]. For instance, Zhong et al. [20] developed a bioinspired wedge-shaped track on a copper substrate, where droplets achieved an average speed of 225.2 mm/s over distances exceeding 25 mm, driven by the synergy of Laplace pressure and wettability gradient forces. Their work also indicated that transport performance degrades with increasing track apex angle. Extending this principle, Zhang et al. [21] showed that droplets can not only move rapidly within patterned wedge channels in horizontal orientation but also ascend against gravity, with the maximum climbing volume influenced by the surface inclination and wettability contrast.

While such studies highlight the effect of wedge structures in directing and collecting liquids, the subsequent critical step—the efficient detachment or drainage of the accumulated liquid—remains less explored. In applications like condensation or self-cleaning, timely liquid removal is essential for sustaining surface performance [24,25,26]. Conventional upright wedge structures often feature a wide base that promotes liquid accumulation and bridging. This can hinder complete surface renewal and lead to the formation of stagnant liquid pools, especially near hydrophobic boundaries [27] or in dense arrays [28]. Alternative approaches, such as wedge-shaped micropillar arrays on superhydrophobic surfaces, promote droplet jumping via coalescence [29,30] but face challenges related to fabrication complexity and the durability of the superhydrophobicity, which, if compromised, leads to deleterious droplet pinning.

Conversely, the “inverted wedge” geometry presents a promising drainage strategy. For instance, Liu et al. [31] utilized multiple slender upright wedge-shaped hydrophilic channels for rapid fog collection and directional convergence, followed by an inverted wedge-shaped drainage ditch. However, due to the ditch’s relatively large apex angle and width, a portion of the liquid was drawn upward against gravity via a Laplace pressure mechanism—similar to a conical Chinese brush drawing ink—resulting in residual liquid and incomplete drainage. This phenomenon has also been reported in reference [28]. However, Liang et al. [32] conducted condensation experiments on patterned surfaces using slender inverted-wedge superhydrophilic channels with a small apex angle. The small apex angle resulted in a limited hydrophilic area at the narrow channel terminus. This configuration promoted rapid and more complete droplet detachment, as gravity drove the coalescence of liquid films to form droplets at specific locations and overcame resistance to enable droplet shedding. They reported a droplet departure frequency nearly four times higher than that of comparable upright wedges. This mechanism also allowed sliding droplets to sweep away adjacent droplets on hydrophobic regions, enabling rapid surface refreshment.

However, previous studies on droplet detachment in inverted wedge-shaped hydrophilic channels are limited in geometric and wettability variations, leaving a lack of systematic and quantitative insight into detachment dynamics. To bridge this gap, we conduct a comprehensive experimental investigation of droplet behavior within laser-etched superhydrophilic channels on hydrophobic/superhydrophobic substrates. By systematically varying channel upper/lower base width, height, and apex angle during controlled water injection, we quantify their effects on droplet formation position, detachment diameter, and formation/detachment frequencies. Furthermore, we identify distinct detachment mechanisms and clarify the role of substrate wettability. This study provides fundamental understanding of droplet dynamics in confined wedge geometries and establishes practical design guidelines for surfaces capable of efficient autonomous liquid drainage, with potential applications in thermal management and microfluidics.

## 2. Materials and Methods

### 2.1. Materials

Analytically pure acetone and ethanol (Macklin Inc., Shanghai, China) were employed for cleaning purposes on highly polished silicon wafers (surface roughness below 0.5 nm). The chlorine-terminated polydimethylsiloxane with a molecular weight ranging from 2000 to 4000 g/mol (Macklin Inc.) and the commercial automotive mirror water repellent “Glaco Mirror Coat Zero” (Soft99 company, Osaka, Japan) were employed for fabricating hydrophobic and superhydrophobic coatings on silicon wafer surfaces, respectively. De-ionized water was homemade, utilizing a deionizer manufactured by Ulupure Inc. (Chengdu, China).

### 2.2. Surface Fabrication

**Figure 1:**
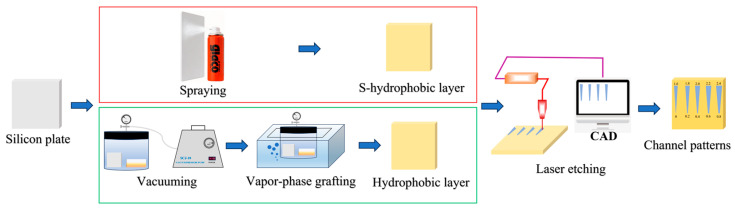
Schematic diagram of fabrication process of the channel patterns.

As illustrated in Figure 1, polished silicon substrates were sequentially cleaned by ultrasonic rinsing in acetone, ethanol, and deionized water, each for 5 min, and then dried with nitrogen. To fabricate a superhydrophobic surface, the “Glaco Mirror Coat Zero” water repellent was uniformly sprayed onto the dried substrates and left to stand for 12 h, resulting in a coating with a micro/nano-structured morphology. For the hydrophobic coating, the dried samples were vertically placed in a glass jar containing a Petri dish with 0.2 mL of chlorine-terminated polydimethylsiloxane. The jar was then evacuated to approximately 0.15 Torr using a vacuum pump and subsequently immersed in a water bath at 60 °C for 1 h [33], thereby forming a hydrophobic layer. To create superhydrophilic channels on these (super)hydrophobic surfaces, predefined channel patterns were lightly etched using a UV laser etcher (Sholaser Corp., Suzhou, China) [32]. This process removed the (super)hydrophobic layer within the channels and concurrently increased the surface roughness of the exposed regions.

### 2.3. Characterization

**Figure 2:**
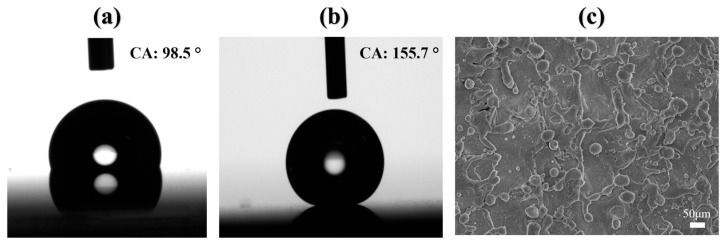
Characterization of (super)hydrophobic surface before and after etching: (**a**,**b**) contact angles of hydrophobic and superhydrophobic surfaces; (**c**) FE-SEM image of channel after etching.

The static water contact angle (CA) of each substrate was measured at room temperature using a DSA25B goniometer (KRÜSS, Hamburg, Germany) prior to laser etching. 2 μL deionized water droplets were deposited at five predefined locations (center and four corners) on every sample surface. The reported CA values represent the average of five independent measurements, with the standard deviation indicating the measurement variability. As shown in Figure 2a,b, the hydrophobic coating exhibited a static CA of 98.5° ± 2.6°, whereas the superhydrophobic coating showed a significantly higher CA of 155.7° ± 2.4°.

The morphology of the laser-ablated channels was examined using a Regulus8100 field-emission scanning electron microscope (Hitachi, Tokyo, Japan). Figure 2c reveals that the laser etching process generated irregular micro- and nanostructures, attributable to localized melting and resolidification. This altered surface topography enabled complete droplet spreading within the channels, resulting in superhydrophilicity.

### 2.4. Experimental Setup and Procedure

**Figure 3:**
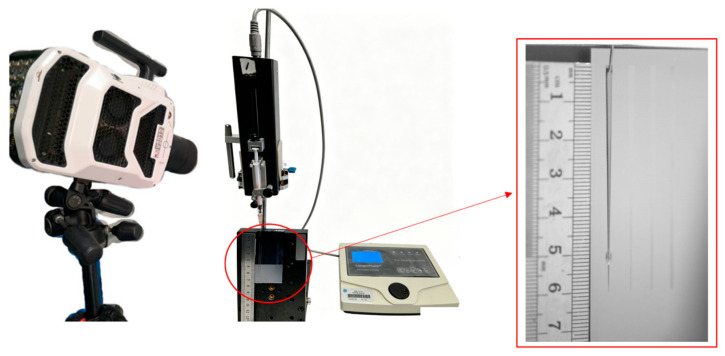
Experimental setup for water injection into the channels.

Figure 3 illustrates the experimental setup for water injection into the channels. A (super)hydrophobic silicon wafer patterned with inverted wedge-shaped superhydrophilic channels of varying dimensions, along with a ruler, was mounted onto a tilt-adjustable stage. Deionized water was injected into each channel at different flow rates using a TS-2A syringe pump (Longer Precision Pump Co., Baoding, China) to simulate various heat fluxes in condensation. Simultaneously, a Phantom v2012 high-speed camera (Vision Research Inc., Wayne, NJ, USA) was employed to record the injection process at a frame rate of 100 fps, capturing detailed fluid dynamic behavior on the surface.

**Figure 4:**
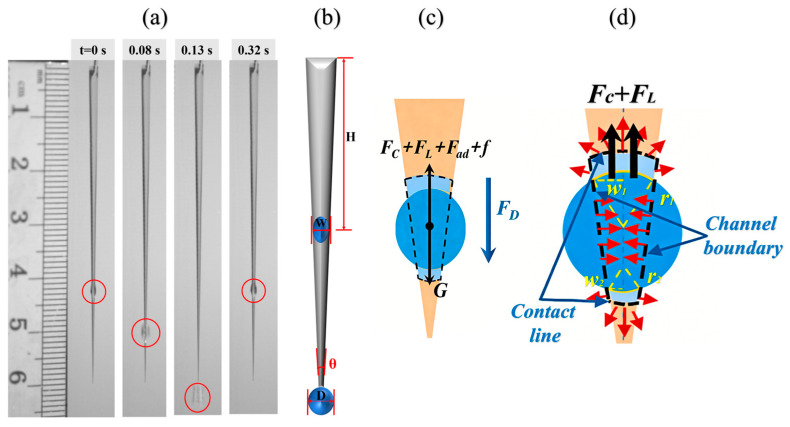
Schematic of parameters and mechanics analysis: (**a**) droplet dynamic cycle of formation, sliding, and detachment; (**b**) important parameters; (**c**) mechanics analysis during droplet descent; (**d**) capillary and Laplace force analysis [34].

Most droplets inside the channel undergo a dynamic cycle, as illustrated in Figure 4a. Upon water injection through the needle, a thin liquid film first coats the channel. As the water volume increases, a droplet forms at a specific site via accumulation of the liquid film. This droplet then slides downward along the channel until it eventually detaches completely from the channel bottom. Following detachment, a new droplet regenerates, repeating the periodic process.

Multiple channel patterns were etched onto each silicon wafer to facilitate comparisons under controlled variables, such as constant area, lower base width, or wedge apex angle. The patterned wafer was secured on the vertical stage, and the syringe pump needle was precisely positioned at the top of the inverted wedge channel. Deionized water was subsequently injected at constant flow rates of 0.1, 0.3, and 0.5 mL/min, with each flow rate maintained for 1 min under ambient conditions of 24 ± 1 °C and 60 ± 4% relative humidity. This procedure was repeated sequentially for adjacent flow channels. Throughout the process, the high-speed camera synchronously recorded the complete evolution of the droplet—from formation and growth to detachment—for subsequent mechanics analysis and parameter comparison.

### 2.5. Experimental Parameters

The geometric parameters of the inverted wedge-shaped channels, specifically the upper base, lower base, and height, were systematically investigated. A specific nomenclature was adopted for channel identification: the code begins with the letter ‘C’, followed by the upper base, lower base, and height, separated by slashes. For instance, the designation C2.0/0/60 corresponds to a channel with an upper base of 2.0 mm, a lower base of 0 mm, and a height of 60 mm. In the subsequent results and discussion, to compare the variations in the top and bottom widths at the same height, only their values are explicitly given, e.g., C2.0/0, while in the x-axis labels of some figures, this is further abbreviated to the form 2.0/0.The regions outside the channels were maintained as superhydrophobic, except for the wettability comparison experiments, where hydrophobic and superhydrophobic coated plates were placed on both sides of a ruler for intuitional comparison.

Key dependent variables were extracted from the video recordings for quantitative analysis. As illustrated in Figure 4b, the droplet formation position (H) is defined as the vertical distance between the center of the droplet and the upper base of the channel at the moment of droplet formation. The parameter (W) denotes the lateral width of the channel at the formation position, while the detachment diameter (D) represents the transverse width of the droplet at the instant it completely detaches from the channel tip. “θ” is the wedge apex angle of the channel. The measurement of these parameters, along with the calculation of droplet formation and detachment frequencies, was performed using the built-in image processing software of the Phantom high-speed camera.

For each experimental condition (channel geometry and flow rate), key parameters were measured over at least five consecutive droplet cycles. The reported values are the averages of these measurements. To ensure measurement accuracy, a ruler was placed adjacent to the test surface in the field of view. The pixel-to-millimeter ratio was calibrated for each video sequence using this ruler prior to measurement. For repeated measurements of the same distance, a deviation on the order of 0.1 mm was occasionally observed. Consequently, the relative uncertainty in measuring the droplet formation position (H) was estimated to be 0.2–1.0%, while that for the detachment diameter (D) ranged from 2.9% to 4.8%. To mitigate this, each parameter was measured multiple times per cycle, and the average was used for that cycle. A recording rate of 100 frames per second (fps) was used, providing a temporal resolution of 0.01 s per frame. This was sufficient to clearly capture the precise moments of formation and detachment, and the associated uncertainty in frequency measurement is considered negligible for the discussed phenomena.

## 3. Results and Discussion

### 3.1. Droplet Mechanics Analysis During Descent in Brief

Droplet mechanics analysis during descent on a surface with wedge-shaped structures is demonstrated in Figure 4c; the force acting on the droplet can be expressed as:

(1) $$F_{D}=F_{g}-F_{C}-F_{L}-F_{ad}-f$$

where *F_D_* denotes the driving force that promotes droplet sliding, *F_g_* denotes gravity, *F_C_* represents capillary forces, *F_L_* denotes the force generated by the Laplace pressure, *F_ad_* denotes the interfacial viscous resistance within the channel, and *f* represents the friction force on the hydrophobic or superhydrophobic region during sliding.

The gravity force *F_g_* is expressed as:

(2) $$F_{g}=\rho Vgsin\beta$$

where *ρ* is the density of water, *V* is the volume of the droplet, *g* is the gravitational acceleration, and *β* denotes the tilt angle of the substrate, which takes a value of 90° in the vertical orientation.

Laplace force (*F_L_*) arising from curvature difference can be expressed as [19,34]:

(3) $$F_{L}\sim \gamma (\frac{1}{r_{2}}-\frac{1}{r_{1}})\frac{sin\theta }{w_{1}-w_{2}}V$$

where *γ* represents the surface tension, *r*_1_ and *r*_2_ represent the local radius of the three-phase contact line between the two sides of the droplet and the surface, *w*_1_ and *w*_2_ are the half-widths of the channels at the wide and narrow ends of the contact line, respectively (shown in Figure 4d), *θ* is the apex angle of the wedge pattern, and *V* is the droplet volume.

The capillary force *F_C_* on the droplet can be obtained by taking the axial derivative of total system surface energy, as shown below [34,35]:

(4) $$F_{C}=\frac{d}{dx}\left(r_{SL}A_{SL}+\gamma_{LG}A_{LG}+\gamma_{SG}A_{SG}\right)$$

Among them, *γ_SL_, γ_LG_,* and *γ_SG_* represent the surface tension of the solid–liquid, liquid–gas, and solid–gas interfaces, respectively, and *A_SL_*, *A_LG_*, and *A_SG_* represent the contact area of the solid–liquid, liquid–gas, and solid–gas interfaces. The variable *x* denotes the displacement of the contact line along the substrate. Capillary forces are considerably weaker than Laplace forces [34,36].

The force *F_ad_* due to viscous dissipation is given by [17]:

(5) $$F_{ad}=\eta A\frac{v}{d}$$

where *η* is the liquid viscosity, *A* is the contact area between the droplet and the channel, *v* is sliding velocity, and *d* is the thickness of the droplet.

The friction force on the hydrophobic or superhydrophobic region during sliding is expressed as:

(6) f=μN

where *μ* is the friction coefficient of the hydrophobic or superhydrophobic region and *N* is the contact force between the droplet and the substrate.

Mechanical analysis reveals that gravity serves as the only driving force for droplet motion. During the droplet descent, continuous water injection leads to an increase in droplet volume, which correspondingly enhances gravity force. Furthermore, the wettability difference in the surface influences both Laplace pressure and capillary forces by altering the surface tension difference. During droplet sliding, the contact area between the droplet and the channel significantly influences the adhesive force. Therefore, minimizing the contact area with the hydrophilic channel can effectively reduce adhesion. Superhydrophobic surfaces exhibit a lower friction coefficient compared to hydrophobic surfaces, leading to reduced frictional resistance during sliding. It should be noted that the present study provides only a preliminary mechanical analysis to reasonably explain the observed droplet dynamics mentioned above, while a more detailed quantitative investigation will be conducted in future work.

### 3.2. Droplet Dynamic Behaviors in Constant-Area Channels

To investigate the influence of channel geometry parameters on droplet detachment, a series of comparative experiments was conducted with one variable held constant. At first, the area of the flow channel was maintained constant while the lower base width was varied, thereby altering the wedge apex angle (θ). The channel heights were set to 20, 40, and 60 mm. For channels with the identical areas, five distinct geometric configurations were designed, with the upper and lower base widths being 2.0/0, 1.8/0.2, 1.6/0.4, 1.4/0.6, and 1.2/0.8 mm, respectively. The formation position of droplets (H), along with the corresponding lateral width (W) and detachment diameter (D), was observed and measured. Subsequently, the frequencies of droplet formation and detachment were calculated.

**Figure 5:**
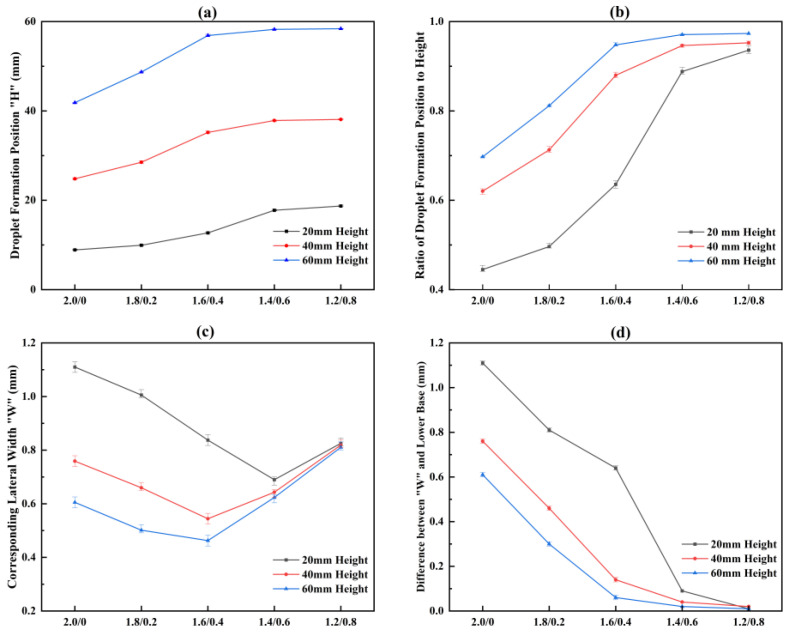
Droplet formation positions and corresponding lateral widths of different channel patterns with the same area: (**a**) droplet formation position; (**b**) ratio of droplet formation position to height; (**c**) corresponding lateral width; (**d**) difference between width and lower base.

During the experiment, we found that the droplet formation position (DFP) inside the channel does not shift with changes in water flow rate. However, in inverted wedge-shaped channels with different heights, the DFP exhibits a consistent trend as the lower base width increases. As shown in Figure 5a,b, in channels with the same area, the DFP gradually approaches the bottom as the lower base width increases; in the C1.4/0.6 and C1.2/0.8 configurations, droplets predominantly form near the bottom. Furthermore, although Figure 5c shows that the measured lateral width corresponding to the DFP initially decreases and then slightly increases with increasing lower base width, the difference between it and the lower base width continues to decrease (Figure 5d). The relationship between the wedge apex angle and the lateral width of the DFP will be further discussed in the subsequent analysis of water injection experiments conducted in identical wedge apex angle channels.

**Figure 6:**
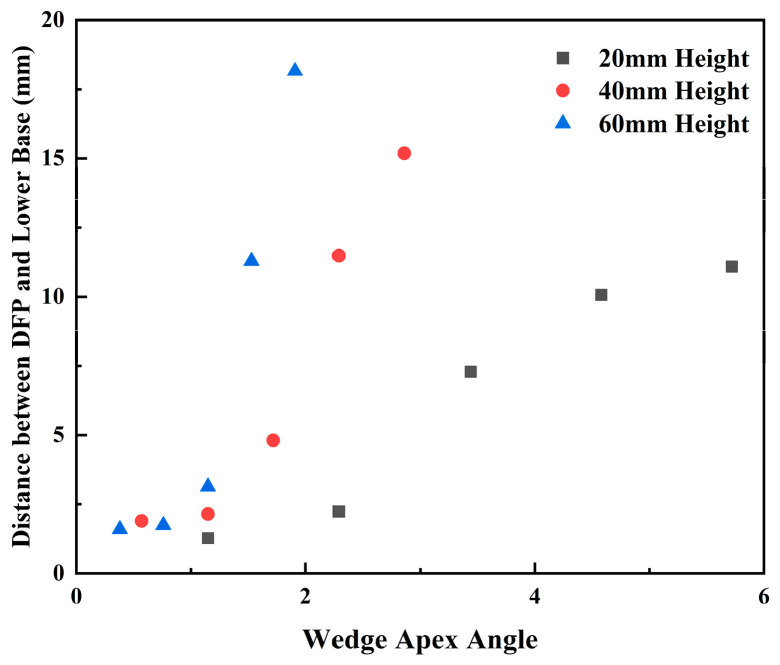
Relationship between wedge apex angle and distance between DFP and lower base.

Although channels of different heights shared the same top and bottom widths in the experiments, their corresponding wedge apex angles differed. To investigate the influence of the wedge apex angle on the DFP, the relationship between the wedge apex angle of each channel and the distance from its DFP to the bottom (i.e., the droplet sliding distance within the channel) is shown in Figure 6. For channels of all three heights, a smaller wedge apex angle resulted in a DFP closer to the bottom. Specifically, when the wedge apex angle was less than 1° (e.g., configurations C1.2/0.8 and C1.4/0.6), droplets formed predominantly near the bottom. In contrast, when the wedge apex angle exceeded 4° (e.g., configurations C2.0/0/20 and C1.8/0.2/20), droplets could form in the upper half of the 20 mm- high channels. Therefore, for fixed-height channels with the same area, a larger wedge apex angle (i.e., a smaller bottom width) is required to achieve a higher DFP, thereby optimizing droplet detachment behavior.

**Figure 7:**
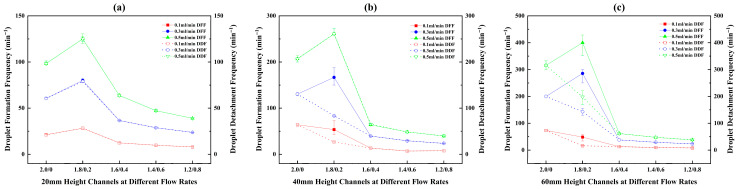
Droplet formation and detachment frequencies within different height channels with the same area: (**a**) 20 mm height; (**b**) 40 mm height; (**c**) 60 mm height.

Based on the analysis of DFPs, it is evident that the initial conditions set by the DFP critically influence the subsequent droplet motion, thereby determining the resultant formation/detachment frequencies and the dominant detachment mechanism. Hence, the droplet formation frequency (DFF) and detachment frequency (DDF) within various channels at different flow rates were obtained through image processing calculations, as presented in Figure 7. In Figure 7a, for different patterned channels with a height of 20 mm, the measured DFF and DDF are nearly identical. This indicates a sequential cyclic process of droplet formation, sliding, growth, and detachment from the bottom, and then the next droplet forms at the same site.

However, in Figure 7b,c, a discrepancy between DFF and DDF is observed in the C1.8/0.2 configuration within the channels with 40 mm and 60 mm heights. For the C1.8/0.2/40 channel at flow rates of 0.1 mL/min and 0.3 mL/min, the DDF is only 50% of the DFF, meaning one detachment occurs for every two droplets formed on average. At 0.5 mL/min, the frequencies become equal again. In the C1.8/0.2/60 channel, the ratios of DDF to DFF at 0.1, 0.3, and 0.5 mL/min are 33.3%, 50%, and 50%, respectively. This implies that 2 to 3 droplets need to form sequentially before a single detachment event occurs.

**Figure 8:**
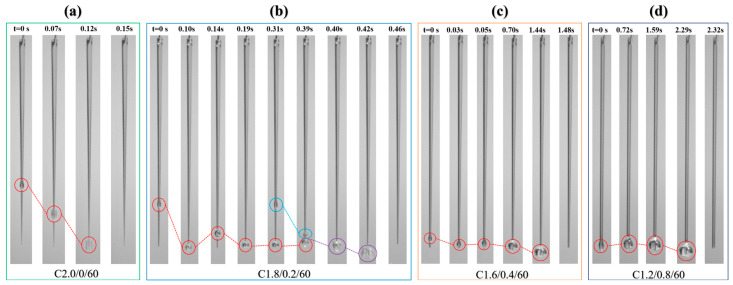
Four mechanisms of droplet behavior across the channels (circles and dotted lines indicate the motion trajectories of droplets; red, light blue, and purple circles represent the first, the second, and the merged droplet after coalescence, respectively): (**a**) Mechanism I: inertia-driven direct shedding; (**b**) Mechanism II: rebound-coalescence-detachment; (**c**) Mechanism III: rebound-accumulation-detachment; (**d**) Mechanism IV: in situ accumulation-growth-detachment.

Close observation of the experimental phenomena reveals that the droplet formation, growth, and detachment behaviors vary across different channels and can be categorized into four main mechanisms, as shown in Video S1 and Figure 8. Mechanism I is inertia-driven direct shedding under gravity (Figure 8a), primarily occurring in C2.0/0 channels. Here, droplets form at a relatively high position and then slide downward driven by gravity. The growing droplet possesses sufficient momentum to overcome the total resistance of the adhesion force (*F_ad_*) from the inherently small and continuously narrowing superhydrophilic channel at the bottom, the very low friction (*f*) of the superhydrophobic surface, as well as Laplace pressure (*F_L_*) and capillary force (*F_C_*), thus detaching directly via inertia.

Mechanism II, rebound-coalescence-detachment, occurs exclusively in C1.8/0.2 channels. As illustrated in Figure 8b, a droplet forming at a relatively high position slides to the bottom but does not detach; instead, it undergoes rapid rebound. After several oscillations with decaying amplitude and velocity, it eventually comes to rest at the bottom. A subsequent droplet then forms, slides down, and coalesces with the first. The merged droplet deforms before final detachment.

Compared to C2.0/0 channels, droplets in C1.8/0.2 form at slightly lower positions, and the superhydrophilic region at the bottom is larger, resulting in stronger adhesion. When a small droplet slides down with low initial momentum, the combined resistance from sliding friction, Laplace pressure, capillary force and adhesive forces can exceed its gravity force, causing rebound. The rebounding droplet is further decelerated by the opposing incoming flow, settles, regrows, and may rebound repeatedly until fully stabilized at the bottom. Detachment ultimately requires the merged droplet volume to reach a critical threshold, aided by the momentum of the last coalescing droplet and the excess kinetic energy released from surface area reduction during coalescence. The number of coalescence events prior to detachment varies with flow rate and height.

Mechanism III is characterized by rebound-accumulation-detachment (Figure 8c), observed in C1.6/0.4 and C1.4/0.6 channels. In these channels, droplets form close to the bottom. After sliding to the bottom, they rebound with minimal amplitude a few times, then continuously accumulate and grow there until reaching a sufficient size for detachment.

Mechanism IV involves droplet detachment after in situ accumulation and growth (Figure 8d). Channels of this type have a very wide bottom. Droplets form, accumulate, and grow directly near the bottom, detaching once they reach a critical size.

As shown in Figure 7, in most cases, the DFF initially increases and then decreases with the increase in the channel’s lower base width, with the peak consistently appearing in C1.8/0.2 channels. Only at the water injection rate of 0.1 mL/min, for channels with heights of 40 mm and 60 mm, the DFF of C2.0/0 is slightly higher than that of C1.8/0.2, causing the overall trend to change to a gradual decrease with increasing bottom width. Among all channels, C1.8/0.2/60 exhibits the highest DFF, reaching 401 min^−1^.

Regarding the DDF, its variation trend does not entirely coincide with that of the DFF due to the droplet rebound and coalescence behavior observed in the C1.8/0.2/40 and C1.8/0.2/60 channels. The trend in some channels shifts from an initial increase followed by a decrease to a gradual decline. The maximum DDF occurs in the C2.0/0/60 channel, reaching 316 min^−1^, while the DDF for the C1.8/0.2/60 channel decreases to 201 min^−1^.

**Figure 9:**
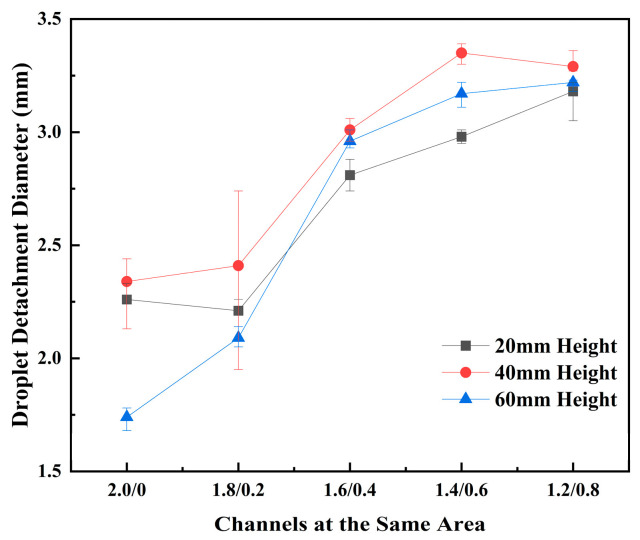
Droplet detachment diameters in all channels during the same-area experiment.

The droplet detachment diameter serves as a key parameter to elucidate the variations in DDF across different channels. As shown in Figure 9, when the lower base width exceeds 0.4 mm, the droplet detachment diameters in all channels are relatively large, concentrated in the range of 2.7 to 3.4 mm. In contrast, for channels with a bottom width of 0–0.2 mm, which exhibit higher detachment frequencies, the detachment diameters are within 2.5 mm and, in some cases, even below 2.0 mm. For instance, in the C2.0/0/60 channel with the highest DDF, the average droplet detachment diameter is only 1.74 mm. The diameter data for the C1.8/0.2/40 channel shows significant scatter. This is because droplet coalescence occurs at flow rates of 0.1 mL/min and 0.3 mL/min, resulting in detachment diameters between 2.57 and 2.74 mm. However, at a flow rate of 0.5 mL/min, the droplet volume and gravity force increase rapidly, enabling inertia-driven direct shedding, and the average detachment diameter drops to as low as 1.94 mm. The DDF under this condition reaches 261 min^−1^, second only to that of the C2.0/0/60 channel (316 min^−1^).

**Figure 10:**
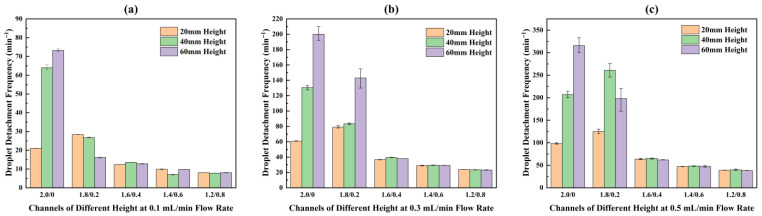
Droplet detachment frequencies in channel patterns of different height at different flow rates: (**a**) 0.1 mL/min; (**b**) 0.3 mL/min; (**c**) 0.5 mL/min.

To systematically investigate the effects of channel height and water injection rate on droplet detachment behavior, the DDFs of channels with different heights at various flow rates are re-plotted in a bar chart format, as shown in Figure 10. A higher flow rate leads to faster droplet growth, resulting in a greater increase in gravity force and momentum and, consequently, an accelerated detachment process. Thus, an increase in the flow rate leads to a consistent elevation in the DDF within all channels.

Regarding the effect of channel height, experimental data show that for all channels with a bottom width greater than 0.4 mm, the DDF differs little across different heights at all flow rates. This is because droplets in such channels primarily form near the bottom and detach via in situ accumulation. Their behavior is mainly governed by the area of the hydrophilic region at the bottom and is insensitive to channel height.

However, significant differences in DDF are observed for C2.0/0 and C1.8/0.2 channels with different heights. For channels like C2.0/0 where detachment relies on inertia-driven sliding, the DFP is crucial. As shown in Figure 6, the droplet sliding distances in the 20 mm, 40 mm, and 60 mm high channels are 11.1 mm, 15.2 mm, and 18.2 mm, respectively. A longer sliding distance allows the droplet to accumulate greater momentum, leading to a higher DDF. This trend becomes more pronounced at higher flow rates. For C1.8/0.2 channels, it was found that the relationship between channel height and DDF is influenced by the flow rate. This is primarily attributed to the droplet rebound behavior observed under some conditions and the varying number of droplets required for coalescence prior to detachment. Overall, a synergistic effect of greater channel height and higher flow rate yields a higher DDF.

### 3.3. Effect of Upper Base Width on Droplet Dynamic Behaviors

Based on previous comparative experiments of channels with the same areas, it has been determined that inverted wedge-shaped channels with a lower base width of 0–0.2 mm exhibit superior droplet detachment performance, and a channel height of 40 mm or 60 mm is preferable. To further investigate the effect of the upper base width on droplet formation and detachment behavior, a subsequent series of experiments was conducted with the channel height fixed at 40 mm and the lower base width fixed at 0 mm, while the upper base width was systematically varied from 1.2 mm to 2.0 mm.

**Figure 11:**
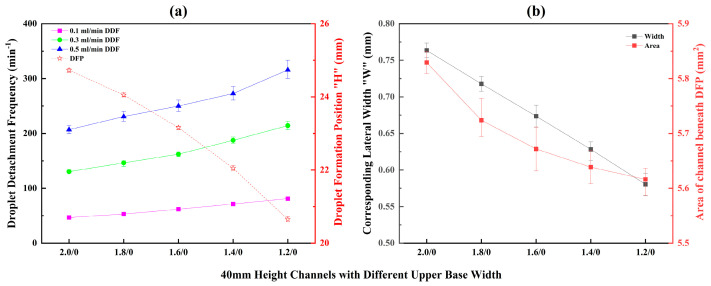
Droplet formation and detachment parameters in channels with different upper base width: (**a**) DDF and DFP; (**b**) corresponding lateral position and area of channel beneath DFP.

The corresponding DFPs and DDFs are shown in Figure 11a. Under all flow rates, the DDF increases as the top width decreases. Channel C1.2/0/40 exhibited the maximum DDF of 318 min^−1^ at a flow rate of 0.5 mL/min. This phenomenon can be analyzed by considering the changes in the DFP, its corresponding lateral width, and the channel area beneath the droplet. As the top width decreases, the DFP moves closer to the upper base (i.e., resides at a higher position), and its corresponding lateral width decreases almost linearly, leading to a reduction in the channel area beneath the DFP (Figure 11b). Consequently, when a droplet starts from a higher initial position and slides down a narrower hydrophilic channel, it can more easily achieve a greater sliding velocity, resulting in a higher DDF. This result demonstrates the advantage of a narrower upper base width for inertia-driven droplet detachment when the lower base width is 0 mm.

### 3.4. Droplet Dynamic Behaviors in Channels with Constant Wedge Apex Angle

**Figure 12:**
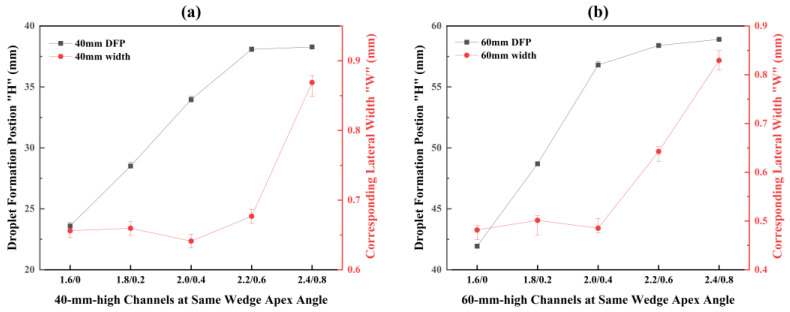
DFP and corresponding later width in different height channels with the same wedge apex angle: (**a**) 40 mm-high channels; (**b**) 60 mm-high channels.

To further investigate the influence of the wedge apex angle, experiments were conducted under a constant wedge apex angle while simultaneously varying the top and bottom widths of the channels. Two channel heights, 40 mm and 60 mm, were selected, with upper/lower base width combinations of 1.6/0, 1.8/0.2, 2.0/0.4, 2.2/0.6, and 2.4/0.8 mm. The DFPs and their corresponding lateral widths for each channel are shown in Figure 12.

As the lower base width increases, the DFP gradually approaches the bottom. The corresponding lateral width “W” remains nearly constant for channels with narrower bottoms, then increases progressively with a further increase in bottom width. For instance, in the first four configurations (lower base width less than 0.6 mm) of the 40 mm-high channels, this lateral width remains stable at 0.65 ± 0.02 mm. However, given that the bottom width in the C2.4/0.8 channel is 0.8 mm, the parameter “W” must exceed the threshold of 0.65 mm and consequently reaches 0.87 mm. Channels with the height of 60 mm exhibit the same trend.

Combined with prior data for equal-area channels with different wedge apex angles, the results indicate that “W” is governed by both the wedge apex angle and the lower base width. When the lower base width is below the characteristic width threshold for a given angle, “W” reduces as the wedge apex angle decreases. For a fixed angle, “W” remains constant across all channels whose lower width is below this angle-specific threshold and increases once the lower width exceeds it.

**Figure 13:**
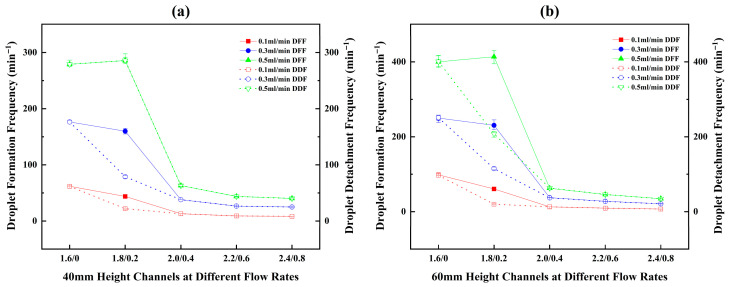
Droplet formation and detachment frequencies in different height channels with the same wedge apex angle. (**a**) 40 mm height; (**b**) 60 mm height.

For channels with the same wedge apex angle, the phenomenon of DDF being lower than DFF is also exclusive to the C1.8/0.2 channels, as demonstrated in Figure 13. The variation trends of DFF and DDF are similar to those obtained in prior experiments on equal-area channels. However, DFF and DDF values of the C1.6/0 channels are generally higher than those of the C1.8/0.2 channels, which stands in contrast to the previous finding that the DFF of the C2.0/0 channel was typically lower than that of the C1.8/0.2 channels. These results further confirm that reducing the upper base width, lower base width, or both, of the inverted wedge-shaped channel contributes to enhanced droplet detachment performance.

### 3.5. Effect of Substrate Wettability on Droplet Detachment

**Figure 14:**
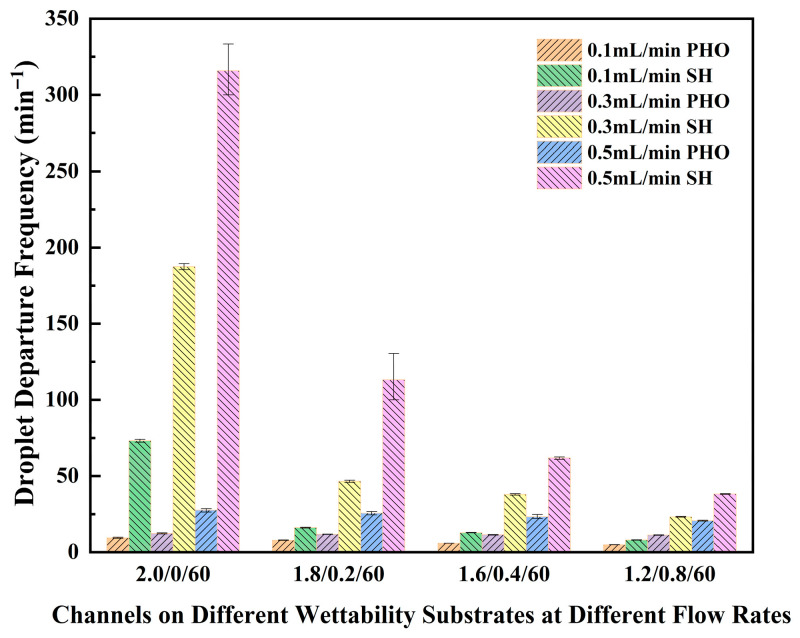
Droplet detachment frequencies in channels on different wettability substrates at different flow rates.

To analyze the effect of substrate wettability on droplet detachment, four types of channels—C2.0/0/60, C1.8/0.2/60, C1.6/0.4/60, and C1.2/0.8/60—representing four distinct droplet detachment mechanisms were fabricated on a hydrophobic surface. The droplet dynamics in these channels were observed, and the DDF was measured at different water flow rates. Similar to previous experiments, droplets formed at different locations within the four channels. In the C2.0/0/60 and C1.8/0.2/60 channels, droplets slid downward at relatively low speeds and subsequently detached either independently or after coalescing with following droplets near the channel bottom. It is worth noting that no droplet rebound was observed in the C1.8/0.2/60 channel. In contrast, in the C1.6/0.4/60 and C1.2/0.8/60 channels, droplets formed near the bottom, grew gradually, and then detached, consistent with the previously described Mechanism IV. The DDF values on the hydrophobic substrate were generally low for all four channel types and exhibited a decreasing trend as the flow rate decreased or the bottom width increased. A comparison with the DDF on the superhydrophobic substrate is presented in Figure 14. Under various flow rates and channel geometries, the ratio of DDF on the superhydrophobic substrate to that on the hydrophobic substrate ranged from 1.65 to 15.3. The most significant difference was observed in the C2.0/0/60 channel, with the maximum ratio occurring at a flow rate of 0.3 mL/min. For the other channels, the ratio remained below 4.5.

**Figure 15:**
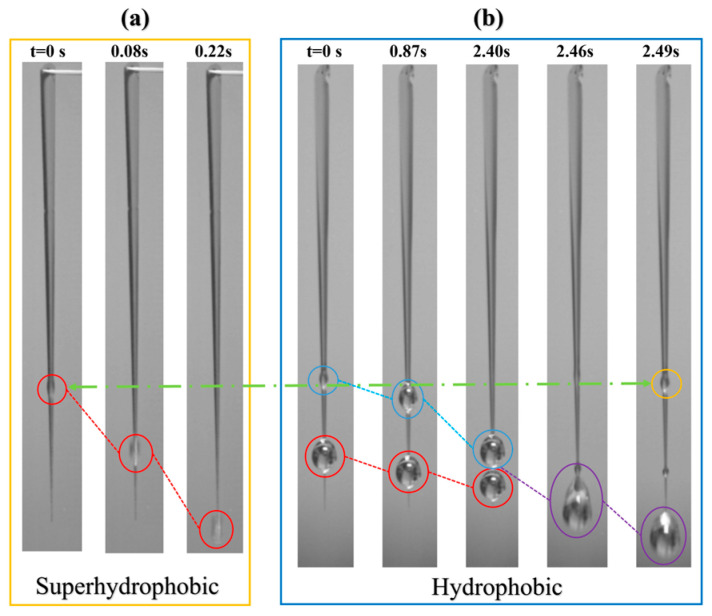
Droplet behaviors in the channels on substrates with different wettability (the circles and dotted lines show the motion trajectories of the droplets: the first (red), the second (light blue), the merged (purple), and the third (orange), respectively; the green dashed arrows mark their initial formation positions): (**a**) superhydrophobic substrate; (**b**) hydrophobic substrate.

A comparative experiment was also conducted by simultaneously injecting water into C2.0/0/60 channels on both a hydrophobic and a superhydrophobic substrate at a flow rate of 0.3 mL/min, as shown in Video S2 and Figure 15. In Figure 15, it was found that the DFPs inside the channels on both surfaces were nearly identical (indicated by the green horizontal dashed arrow). However, the droplet detachment speed was significantly faster on the superhydrophobic surface. On the superhydrophobic surface, the time from droplet formation to complete detachment was only 0.22 s. In contrast, on the hydrophobic surface, after the first droplet (red circle) grew to a certain size, it took 2.49 s and required coalescence with the following droplet (light blue circle) to achieve a single detachment event.

The experimental results indicate that the wettability of the external substrate does not affect the DFP but primarily influences the speed at which the droplet slides down the channel after formation. This can be attributed to the differences in the friction coefficient and the solid–liquid contact area during sliding.

Specifically, on a superhydrophobic substrate, the lower friction coefficient and the pronounced wettability contrast with the superhydrophilic channel enable the droplet to slide with a minimal contact area. This facilitates rapid acceleration, allowing the droplet to detach via an inertia-driven direct shedding mechanism. In contrast, on a hydrophobic substrate, the droplet can spread more over the hydrophobic region, resulting in a larger contact area. Consequently, the droplet slides more slowly due to the higher frictional resistance. Although continuous water injection increases the droplet volume and gravitational force, the concurrently enlarged contact area leads to a proportionally greater resistive force. This often results in a net reduction in sliding speed. Under such conditions, a subsequent droplet can form and catch up before the first droplet detaches. The two droplets then coalesce near the channel bottom, and detachment occurs only after this coalescence event.

Therefore, the low-friction superhydrophobic substrate is essential for achieving inertia-driven direct shedding in channels with a bottom width of zero. This factor is a key reason for the significantly higher DDF observed on such surfaces.

## 4. Conclusions

This study systematically investigates the key factors governing droplet dynamic behaviors within inverted-wedge superhydrophilic channels patterned on (super)hydrophobic substrates. The primary findings and conclusions are summarized as follows:

1. Channel geometry dictates droplet formation and initial behavior. The DFP is primarily determined by the channel’s wedge apex angle and lower base width, independent of the water injection flow rate. For channels with the same area, a smaller bottom width positions the DFP farther from the bottom, providing a longer sliding distance. A critical DFP-corresponding lateral width threshold exists for each wedge apex angle; below this threshold, the width remains constant but increases once the bottom width exceeds it.
2. Four distinct droplet detachment mechanisms are identified. The dominant mechanism shifts progressively from I to IV as the lower base width increases. The highest DDF is achieved by Mechanism I, which is favored in channels with a minimal lower base (~0 mm). In such configurations, droplets form at elevated positions and acquire significant momentum by sliding over an extended distance, further amplified by increased channel height. In contrast, Mechanism II, involving rebound and coalescence (predominant in C1.8/0.2 channels), results in a DDF lower than the DFF. Mechanisms III and IV, occurring with wider lower bases, are characterized by droplet growth near or at the bottom prior to detachment.
3. Best-performing parametric design within the tested window. The most efficient droplet detachment is achieved by employing a slender inverted wedge-shaped channel with a narrow bottom (~0 mm), a low wedge apex angle (narrow upper base), and a sufficient height (≥40 mm), fabricated on a superhydrophobic substrate. This configuration combines a high formation site for momentum gain with minimal sliding resistance, promoting rapid, inertia-driven shedding. Channel C1.2/0/40 exhibited the maximum DDF of 318 min^−1^ at a flow rate of 0.5 mL/min.

This study investigates droplet detachment dynamics in inverted wedge-shaped channels. The results provide an experimental foundation and preliminary design principles for developing passive droplet control surfaces, with potential applications in condensation enhancement, microfluidic systems, and self-cleaning interfaces. For instance, in condensation surface design, a superhydrophobic substrate patterned with multiple slender inverted-wedge superhydrophilic channels can be employed. In this configuration, droplets can detach under inertia within their respective channels and may also coalesce with droplets from adjacent channels during sliding, leading to cross-channel droplet shedding. Even in designs where droplets tend to remain suspended at the channel bottom, the spacing between neighboring channels can be adjusted to allow suspended droplets from different channels to contact and achieve coalescence-induced detachment. Future work will include experimental validation under practical condensation conditions, refinement of the droplet dynamics model, and further extension of the applicability of the findings.

## Data Availability

The original contributions presented in this study are included in the article. Further inquiries can be directed to the corresponding author.

## Supplementary Materials

The following supporting information can be downloaded at: https://www.mdpi.com/article/10.3390/mi17050575/s1, Video S1: Four distinct droplet detachment mechanisms; Video S2: Droplet detachment behaviors on different wettability substrates.

## Abbreviations

The following abbreviations are used in this manuscript:

DFP	Droplet Formation Position
DFF	Droplet Formation Frequency
DDF	Droplet Detachment Frequency
